# Towards the world we want

**DOI:** 10.2471/BLT.14.144808

**Published:** 2014-09-01

**Authors:** Oleg Chestnov, Werner Obermeyer, Joy St John, Menno Van Hilten, Alexey Kulikov

**Affiliations:** a World Health Organization, Geneva, Switzerland.; b World Health Organization, 1 Dag Hammarskjold Plaza, 885 2^nd^ Ave, New York, NY 10017, United States of America.

Globalization offers great opportunities, but its benefits are at present very unevenly shared. Inequity in health at the population level is affected by global changes in marketing and trade, rapid urbanization and population ageing. The social, economic and physical environments in low- and middle-income countries often afford their populations much lower levels of protection from the risks and consequences of noncommunicable diseases (NCDs).^1^ Such factors contribute to the 12 million premature deaths and estimated economic losses of US$ 500 billion from NCDs that occur in these countries every year. However, lives and resources can be saved by investing in better prevention, control and treatment measures.^2^

In 2011, the World Health Organization (WHO) was assigned a leadership and coordination role in supporting national efforts to address noncommunicable diseases by the *Political Declaration of the high-level meeting of the General Assembly on the prevention and control of non-communicable diseases*.^3^ Three years later, there is a global road map in place based on nine concrete global targets for 2025, organized around the WHO *Global action plan for the prevention and control of NCDs 2013–2020*. The global action plan, when implemented collectively by Member States, international partners and WHO, will help to attain a global target of a 25% reduction in premature deaths from NCDs by 2025.^4^ The United Nations Interagency Task Force on NCDs, which the United Nations Secretary-General established in July 2013 and placed under the leadership of WHO, has started to provide support to national efforts to build solutions to address NCDs. The WHO Global Coordination Mechanism on NCDs, established in May 2014, will facilitate contributions from non-State actors.

Progress within countries matters most. Some striking achievements emerge from a survey conducted by WHO in 2013. Of the 172 countries reporting data, 95% have a unit or department in the Ministry of Health responsible for NCDs. Half now have an integrated operational plan with a dedicated budget. The number of countries conducting recent surveys of risk factors jumped from 30% in 2011 to 63% in 2013. In other words, more countries are getting the basics in place.

However, in July 2014 at the United Nations General Assembly ministers from across the world found that overall progress is insufficient and highly uneven. The United Nations review saw no lack of commitment, but witnessed a lack of capacity to act, especially in low- and middle-income countries, due to a lack of access to expertise which is only available through international cooperation. To move forward, the outcome document adopted by the United Nations review presents a highly focused agenda for strengthening international cooperation.

The outcome document also contains next priorities in clear steps that will guide action until 2018, when the United Nations General Assembly will convene a third high-level meeting on NCDs. These include five commitments from Member States;^5^ setting national NCD targets for 2025, developing national multisectoral plans and implementing the WHO* Global action plan for the prevention and control of NCDs 2013–2020* to reduce risk factors and strengthen health systems.

WHO has three major assignments; the first is to prepare a framework for country action aimed at supporting national efforts to improve health through action across sectors on risk factors for NCDs. The second is to develop an approach to register and publish contributions of non-State actors to the achievement of the nine global targets and the third is to submit a progress report to the United Nations General Assembly. The Organisation for Economic Co-operation and Development has been tasked with developing a code to track official development assistance for NCDs.

In July 2014, ministers in New York also agreed to give due consideration to addressing NCDs in the elaboration of the post-2015 development agenda, taking into account that NCDs constitute one of the major challenges for development in the twenty first century.^6^ A proposed target to reduce premature mortality from NCDs by one third by 2030 will be considered by Member States in September 2014 at the United Nations General Assembly. This milestone will provide critical guidance to the September 2015 United Nations Summit, which will adopt the post-2015 agenda. The discussions in July 2014 provided a timely opportunity for rallying political support for bolder measures in the post-2015 era.

To build a future in which globalization becomes a positive force for all the world’s peoples, political commitment is needed. Only with such commitment can WHO orchestrate the broad collaboration required to make progress.

## References

[1] Noncommunicable diseases country profiles 2014 [Internet]. Geneva: World Health Organization; 2014. Available from: http://www.who.int/substance_abuse/publications/global_alcohol_report/profiles/en/ [cited 2014 July 16].

[2] Chestnov O, Van Hilten M, McIff C, Kulikov A. Rallying United Nations organizations in the fight against noncommunicable diseases. Bull World Health Organ. 2013;91(9):623–623A. 24101775 10.2471/BLT.13.128348 PMC3790227

[3] Political declaration of the high-level meeting of the General Assembly on the prevention and control of non-communicable diseases (Document A/RES/66.2). In: 2011 High Level Meeting on Prevention and Control of Non-communicable Diseases, New York, 19–20 September 2011. New York: United Nations; 2011. Available from: http://www.un.org/en/ga/ncdmeeting2011 [cited 2014 July 16].

[4] Follow-up to the Political Declaration of the high-level meeting of the General Assembly on the prevention and control of non-communicable diseases (Resolution WHA66/10). In: Sixty-sixth World Health Assembly, Geneva, 27 May 2013. Geneva: World Health Organization; 2013. Available from: http://apps.who.int/gb/ebwha/pdf_files/WHA66/A66_R10-en.pdf [cited 2014 July 16].

[5] Outcome document of the high-level meeting of the General Assembly on the comprehensive review and assessment of the progress achieved in the prevention and control of non-communicable diseases (Resolution A/RES/68/300). In: Sixty-eighth United Nations General Assembly documentation.New York: United Nations; 2014. Available from: http://www.un.org/depts/dhl/resguide/r68_en.shtml [cited 2014 August 18].

[6] Note by the Secretary-General transmitting the report of the Director-General of the World Health Organization on the prevention and control of non-communicable diseases (Document A/68/650). In: Sixty-eighth United Nations General Assembly documentation. New York: United Nations; 2014. Available from: http://www.un.org/ga/search/view_doc.asp?symbol=A/68/650 [cited 2014 July 18].

